# Postmenopausal Breast Cancer in Women, Clinical and Epidemiological Factors Related to the Molecular Subtype: A Retrospective Cohort Study in a Single Institution for 13 Years. Follow-Up Data

**DOI:** 10.3390/ijerph17238722

**Published:** 2020-11-24

**Authors:** Cristina Marinela Oprean, Serban Mircea Negru, Dorel Ionel Popovici, Sorin Saftescu, Robert-Alexandru Han, Gabriel-Mugurel Dragomir, Teodora Hoinoiu, Alis Dema

**Affiliations:** 1 Discipline of Morpho-pathology, “Victor Babeş” University of Medicine and Pharmacy, Eftimie Murgu Sq. No.2, 300041 Timişoara, Romania; cristina.oprean@oncohelp.ro (C.M.O.); dema.alis@umft.ro (A.D.); 2 Department of Oncology—ONCOMED Outpatient Unit, Ciprian Porumbescu Street, No. 59, 300239 Timisoara, Romania; serban.negru@oncohelp.ro (S.M.N.); dorel.popovici@oncohelp.ro (D.I.P.); sorin.saftescu@oncohelp.ro (S.S.); 3 Department of Oncology—ONCOHELP Hospital Timisoara, Ciprian Porumbescu Street, No. 59, 300239 Timisoara, Romania; robert.han@oncohelp.ro; 4 Discipline of Oncology, “Victor Babeş” University of Medicine and Pharmacy, Eftimie Murgu Sq. No.2, 300041 Timişoara, Romania; 5 Department of Teaching Training—POLYTEHNICAL, University of Timisoara, 300223 Timisoara, Romania; mugur.dragomir@upt.ro; 6 Department of Clinical Practical Skills, “Victor Babeş” University of Medicine and Pharmacy, Eftimie Murgu Sq. Nr.2, 300041 Timişoara, Romania

**Keywords:** breast cancer, postmenopausal status, molecular subtype, stage, location, prognostic factor

## Abstract

This study focused on the characteristics of postmenopausal breast cancer in the population of southeastern Europe. This retrospective study explored the clinical, epidemiological, and molecular characteristics of women with postmenopausal breast cancer. Material and methods: A retrospective cohort study was performed on 721 postmenopausal breast cancer patients selected from the database of our institution. The data collected consisted of age, living environment, location of the breast tumor, stage of the disease, and molecular sub-type. Patient characteristics were collected based on a systematic chart audit from medical records. The data were analyzed using SPSS 20.0 and Pearson analysis. Results: The most frequent age range for breast cancer diagnosis was 51 to 70 years old. Most of the patients (80.7%) came from an urban environment. The vast majority of patients were initially diagnosed in stage II (40.3%) and III (30.3%). The most frequent molecular sub-types were luminal B (39%) and luminal A (35.4%). Almost half of the breast tumors were located in the upper outer quadrant (48.8%). Conclusions: The results of this study describe the profile of patients in southeastern Europe within our institution diagnosed with postmenopausal breast cancer. In our study, patients were first diagnosed with more advanced stages of breast cancer compared with other European countries.

## 1. Introduction

Breast cancer is the most common type of cancer in the world, becoming the leading cause of cancer-related deaths among women in the world, as well as in southeastern Europe [1].

The incidence of breast cancer is the highest in economically developed countries. Due to delayed methods of diagnosing breast cancer, we are witnessing an increased incidence of advanced development of this disease in developing and low-income countries [2].

Globally, the incidence of breast cancer has different rates of occurrence [3]. According to GLOBOCAN 2018, 9629 new cases were registered in Romania, ranking second after lung cancer [4].

Age is an important factor in the heterogeneity of breast cancer. Breast cancer in older women appears biologically distinct from breast cancer in younger women [5]. Women have an increased risk of developing cancer as they age [6]. The luminal A breast cancer subtype is encountered more frequently in postmenopausal women [7]. A study conducted in Japan that looked at the prognostic value of KI-67 found that patients over 65 years of age develop tumors with a lower proliferation rate; there was no difference in the KI-67 tumor index for patients aged 36–50 and 50–65 years [8]. In a study focused on the population of breast cancer patients in India, the diagnoses of most (75%) breast cancer patients occurred in stage III. Stage IV disease was diagnosed only in postmenopausal women [9].

Studies have compared the clinical and pathological characteristics of postmenopausal breast cancer patients with those of premenopausal patients, rather than describing them independently.

Data regarding the characteristics of postmenopausal breast cancer in the Romanian population are insufficient. The primary aim of this study was to explore the clinical, epidemiological, and molecular characteristics of the women diagnosed with postmenopausal breast cancer registered in our oncological database. Secondly, we aimed to identify whether a relationship exists between these characteristics.

## 2. Materials and Methods

This study was a retrospective observational study performed in a single institution—a general oncology specialized center.

### 2.1. Patients 

We investigated the medical records of 1000 breast cancer patients registered in the database of our clinic that were admitted between May 2004 and December 2017. A total of 271 patients diagnosed with premenopausal breast cancer and 8 male patients were excluded from our study.

The data of the 721 patients selected, diagnosed with postmenopausal breast cancer and for whom complete clinical records were available, were statistically processed using SPSS 20.0. A trained medical registrar performed data collection between December 2017 and July 2018. To avoid any potential sources of bias, a senior and a junior medical oncologist independently performed a review and a double-check of the accuracy of the collected data. We finalized this process in July 2019. The source documents are the patients’ outpatient paper charts.

Postmenopausal status was defined as physiological or early menopause at the time of each patient’s diagnosis [6]. The main causes for premature menopause were: previous medical conditions, surgical procedures, such as total hysterectomy or bilateral oophorectomy, or pelvic radiotherapy [10].

The Steering Committee of the Outpatient Unit approved this retrospective study in July 2017, the date the study was performed. The retrospective data were collected and reviewed in compliance with the ethical standards set out by the Steering Committee of the institution and with the Declaration of Helsinki. For this retrospective study, the patients’ consent for the review of their medical records was not required by the Steering Committee of the institution.

### 2.2. Data Collection

The epidemiological data that we considered for this study were: age, defined as the age at the time of diagnosis, and living environment (rural or urban). Areas forming settlements with populations of over 10,000 are urban, as defined by the Office for National Statistics urban area boundaries based on land use. The remainder is defined as a rural town and fringe, village, or hamlet and dispersed using detailed postcode data. The clinical data collected were: location of the breast tumor and stage of the disease at the time of diagnosis. The information about the tumor location inside the quadrant (tumor site) was obtained from mammographies, MRI, and breast ultrasounds undergone by the patients during their initial consultation at the time of diagnosis. The clinical data recorded in the oncology outpatient charts or the surgery medical letters were also used as a source of documentation.

TNM classifications, both clinically and/or pathologically, were used for establishing the stage of the disease. For the patients who underwent surgical treatment, the pathological TNM was used; for the remaining patients, we used the clinical TNM. Depending on the year of diagnosis, we used the 5th, 6th, and 7th AJCC TNM classification [11,12,13]. 

The molecular subtype was classified for invasive tumors according to the 2017 St. Gallen Consensus meeting, depending on the ER, PR, KI-67, and HER2 status [14]. According to this classification, patients were divided into five subgroups: luminal A, luminal B, luminal HER- positive, non-luminal HER2-positive, and triple-negative breast cancer. 

These parameters were obtained from the immunohistochemistry (IHC) examination of the breast cancer tissue sample. The tissue was examined in 2–3 external laboratories located in Timisoara, Romania. All these labs used the manual or automatic platforms for the IHC results. ER and/or PR were considered positive when the value was >1%. The cut-off value for KI-67 to define luminal A to luminal B tumors was considered to be 20%. To distinguish HER2-positive from HER2-negative tumors, pathologists performed IHC and in situ hybridization tests and use the ASCO/CAP guidelines valid at the time of the examination. 

### 2.3. Statistical Analysis

The data were statistically processed using SPSS 20.0. Descriptive statistics were used to group subjects by categories such as age, living environment, tumor location, stage of the disease, and molecular subtype. The method of association tables, related to descriptive statistics, was also used. Pearson correlation analysis was used to assess the correlation between various parameters (age and stage, age and molecular subtype, stage and molecular subtype), where *p* < 0.05 was considered to define the statistical significance difference.

## 3. Results

The mean age was 62.6 years, and the age range was 33–87 years. Distribution by age groups is shown in Table 1. Out of a total of 721 patients, the majority of patients, i.e., 581 (80.7%), came from an urban environment and only 139 (19.3%) from a rural area. This information was missing for only one patient (0.1%).

The analysis of the different age groups and molecular subtypes showed that in the youngest age group, i.e., 30 to 40 years, the luminal HER2-positive subtype was prevalent. In the middle age group (41–50 and 51–60 years), the luminal B subtype prevailed. For the next two patient groups, 61–70 and 71–80, most of the patients had the luminal A subtype. The oldest age group, 81–90 years, showed a predominant luminal B subtype. The data are summarized in Table 2.

The correlation between age groups and molecular subtypes showed an inversely proportional relationship between the two variables (r = −0.114*, *p* < 0.01, Table 3).

The majority of patients with stage 0, I breast cancer were in the 61–70 years age group. An initial diagnosis of stage II or III breast cancer prevailed for the 51–60 years age group. Most patients in stage IV were in the 61–70 years age group. The data are summarized in Table 4. Using Pearson correlation, no statistically significant correlation was established between the stage of the disease and age at diagnosis, the significant threshold being *p* = 0.322 (*p* > 0.05; Table 5).

Among the 721 patients selected, 509 (70.6%) were diagnosed in stages II and III. The distribution of patients by stage is summarized in Table 6.

The analysis of the molecular subtype revealed that the most predominant molecular subtype in this postmenopausal population was luminal B, 281 patients (39%), closely followed by luminal A with 255 patients (35.4%). The result of the molecular subtype distribution is shown in Table 7.

The most frequent breast tumor location in this postmenopausal breast cancer population was the Upper-Outer quadrant (UOQ) with 352 patients (48.8%), and the least frequent was the Lower-Inner quadrant (LIQ) with 56 patients (7.8%). The results are summarized in Table 8.

**Figure 1:**
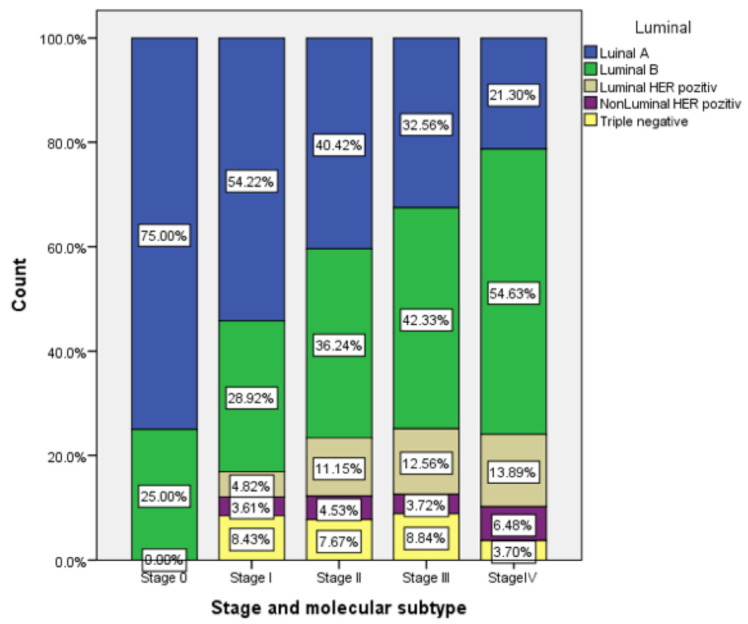
Stage of the disease related to molecular subtype.

This analysis of subtypes showed that the luminal A subtype was prevalent in stages 0, I, and II. The luminal B subtype was prevalent in stages III and IV. The last ones represented in all stages were the HER2-positive and triple negative subtypes. The data are illustrated in Figure 1. The Pearson correlation showed a small and positive correlation coefficient (r = 0.089, *p* < 0.05). The correlation is weak, but it is statistically significant, with the significance threshold of *p* < 0.05 (Table 9).

## 4. Conclusions

This was a retrospective, single-institution study of postmenopausal Romanian women with breast cancer. In this study, most postmenopausal patients diagnosed with breast cancer were between 51 and 60 years of age (35.6%) and 61 and 70 years of age (35.6%). The data are similar to national Romanian statistics [4,15]. The highest incidence rates of breast cancer in Romania occurs in the age group >65 years [16]. Similar data were reported in the U.K. statistics [17].

We found major differences between the urban and rural population, with 80.7% of the patients coming from urban areas and only 19.3% from rural areas. An Australian study showed similar results [18]. These differences can be explained by low access to mammography screening in rural areas, which accounts for determining an advanced stage of disease at diagnosis [19].

Breast cancer was often diagnosed in our study group in stages II (40.3%) and III (30.3%). The diagnosis in stage IV of the disease was found in 15.3% of the cases. Compared with other European statistics, we found a higher incidence in the advanced stages of breast cancer disease and a low percentage of patients diagnosed in stage I (11.5%) [17,20,21].

Regarding the molecular subtype distribution in our study population, the results showed a predominance of the luminal B (39%) and luminal A (35.4%) subtypes. The HER2-positive subtype was found in 15% of the patients, with more luminal HER2-positive (10.7%) than non-luminal HER2-positive (4.3%) cases. The statistical data are similar to those reported in other studies [22].

Looking at the location of the primary tumor, our results showed that the most frequent location was the upper quadrants (65.3%), more in the UOQ (48.8%) than in the UIQ (16.5%). These data are in agreement with the data of the study conducted on 13,984 tumors. The results of this study, carried out using the tumor registry in Tacoma, WA, USA, showed that more than half (58%) of the breast tumors were located in the UOQ. The location of the tumor could be correlated with the survival, prognosis, stage of the disease, and positive axillary lymph nodes. Patients with a localization of the primary tumor in the UOQ have a significantly higher survival rate than women with a tumor in other quadrants [23]. The differences in survival rates depending on the location of the tumor are major, and other prognostic factors must be considered [24].

We found no statistically significant correlation between the stage of breast cancer and age group (*p* > 0.05). In our study, the correlation between the age group and molecular subtype was statistically significant (*p* < 0.01), meaning that the aggressiveness of breast tumors lowered with age. This result is similar to the results of studies conducted in other populations. The American study that investigated age-specific changes in intrinsic breast cancer subtypes in older women showed that age at the time of the diagnosis is not an independent prognostic factor for the outcome [25]. The stage–molecular subtype correlation was weakly positive and statistically significant (*p* < 0.05). The luminal A subtype is mostly associated with early stages (0, I, and II), and luminal B with advanced stages (III and IV). More than half (53.66%) of our stage I patients had a the luminal A subtype. This result is similar to a Chinese retrospective study that concluded that there were significant differences in the distribution of TNM staging among the five subtypes of breast cancer [26].

The results of this study describe the profile of Romanian patients in our institution with postmenopausal breast cancer. This population is rarely described separately from the population of other European countries. Although Romania is representative of middle-income countries, the statistical results of our study are similar to other high-income European countries. The only difference is related to the stage of the disease. The patient population in our study presented with more advanced stages of the disease at diagnosis. Further prospective studies should be conducted to validate these results.

## Figures and Tables

**Table 1:** Patient distribution by age group.

Age at Diagnosis	Patients N = 721	Percent %
0–40 years	7	1.0
41–50 years	50	6.9
51–60 years	257	35.6
61–70 years	257	35.6
71–80 years	129	17.9
81–90 years	21	2.9
Total	721	100.0

**Table 2:** Age groups and luminal subtypes.

		Molecular Subtype					
Age at Diagnosis		Luminal A	Luminal B	Luminal HER-Positive	Non-Luminal HER-Positive	Triple Negative	Total
30–40 years	N	2	1	3	0	0	6
	%	33.3%	16.7%	50.0%	0.0%	0.0%	100.0%
41–50 years	N	12	21	9	3	2	47
	%	25.5%	44.7%	19.1%	6.4%	4.3%	100.0%
51–60 years	N	76	108	28	12	26	250
	%	30.4%	43.2%	11.2%	4.8%	10.4%	100.0%
61–70 years	N	105	96	29	8	16	254
	%	41.3%	37.8%	11.4%	3.1%	6.3%	100.0%
71–80 years	N	54	48	5	7	7	121
	%	44.6%	39.7%	4.1%	5.8%	5.8%	100.0%
81–90 years	N	6	7	3	1	1	18
	%	33.3%	38.9%	16.7%	5.6%	5.6%	100.0%
Total	N	255	281	77	31	52	696
	%	36.6%	40.4%	11.1%	4.5%	7.5%	100.0%

**Table 3:** Correlation between age at diagnosis and molecular subtype.

Correlations			
		Age at Diagnosis	Molecular Subtype
Age at Diagnosis	Pearson Correlation	1	−0.114 *
	Sig. (2-tailed)		0.003
	N	721	696
Luminal	Pearson Correlation	−0.114 **	1
	Sig. (2-tailed)	0.003	
	N	696	696

* The correlation is significant at the 0.01 level (2-tailed). ** The correlation is significant at the 0.01 level (2-tailed).

**Table 4:** Stage related to age.

Stage		Age at Diagnosis (years)						Total
		30–40	41–50	51–60	61–70	71–80	81–90	
0	N	0	0	1	2	1	0	4
	%	0.0%	0.0%	25.0%	50.0%	25.0%	0.0%	100.0%
I	N	0	2	28	38	13	2	83
	%	0.0%	2.4%	33.7%	45.8%	15.7%	2.4%	100.0%
II	N	2	23	105	103	53	5	291
	%	0.7%	7.9%	36.1%	35.4%	18.2%	1.7%	100.0%
III	N	3	14	81	69	41	10	218
	%	1.4%	6.4%	37.2%	31.7%	18.8%	4.6%	100.0%
III	N	3	14	81	69	41	10	218
	%	1.4%	6.4%	37.2%	31.7%	18.8%	4.6%	100.0%
IV	N	1	10	37	42	17	3	110
	%	0.9%	9.1%	33.6%	38.2%	15.5%	2.7%	100.0%
Total	N	6	49	252	254	125	20	706
	%	0.8%	6.9%	35.7%	36.0%	17.7%	2.8%	100.0%

**Table 5:** Correlation between the stage of the disease and age at diagnosis.

Correlations			
		Patients N	Age at Diagnosis
Stage	Pearson Correlation	1	−0.037
	Sig. (2-tailed)	-	0.322
	N	706	706
Age at Diagnosis	Pearson Correlation	−0.037	1
	Sig. (2-tailed)	0.322	
	N	706	721

**Table 6:** Stage of the disease at diagnosis.

Correlations			
	Stage of the Disease	Patients, N	Percent, %
Stage	0	4	0.6
	I	83	11.5
	II A	171	23.7
	II B	120	16.6
	III A	87	12.1
	III B	111	15.4
	III C	20	2.8
	IV	110	15.3
	**Total**	706	98
Not evaluable		15	2.0
Total		721	100.0

**Table 7:** Molecular subtypes of breast cancer.

Molecular Subtype		Patients, N	Percent, %
Stage	Luminal A	255	35.4
	Luminal B	281	39.0
	Luminal HER-positive	77	10.7
	Non-Luminal HER-positive	31	4.3
	Triple negative	52	7.2
	Total	696	96.5
Not Evaluable		25	3.5
Total		721	100.0

**Table 8:** Tumor location in the breast.

Tumor Location, Breast Quadrant **		Patients, N	Percent, %
	LOQ	74	10.3
	UOQ	352	48.8
	CQ-	94	13.0
	LIQ	56	7.8
	UIQ	119	16.5
	BB	7	1.0
	WB	11	1.5
	Total	713	98.9
Not evaluable		8	1.1
Total		721	100.0

** Breast Quadrant: LOQ—Lower-Outer Quadrant, UOQ—Upper-Outer Quadrant, CQ—Central Quadrant, LIQ—Lower-Inner Quadrant, UIQ—Upper-Inner Quadrant, BB—bilateral breast, WB—whole breast.

**Table 9:** Correlation between stage and molecular subtype.

Correlations			
		Molecular Subtype	Stage of the Disease
Luminal	Pearson Correlation	1	0.089
	Sig. (2-tailed)		0.019
	N	696	689
Stage of the disease	Pearson Correlation	0.089	1

## Abbreviations

ER—Estrogen receptor; PR—Progesterone receptor; KI-67—a cell proliferation-associated antigen; MRI—magnetic resonance imaging; TNM—tumor (T), nodes (N), metastases (M); AJCC—American Joint Committee on Cancer; IHC—Immunohistochemistry; SPSS—Statistical Package for the Social Sciences; LOQ—Lower-Outer Quadrant; UOQ—Upper-Outer Quadrant; CQ—Central Quadrant; LIQ—Lower-Inner Quadrant; UIQ—Upper-Inner Quadrant; BB—bilateral breast; WB—Whole Breast.
